# Catechol-O-Methyltransferase Val158Met Polymorphism Is Associated with Somatosensory Amplification and Nocebo Responses

**DOI:** 10.1371/journal.pone.0107665

**Published:** 2014-09-15

**Authors:** Laura Wendt, Antje Albring, Sven Benson, Harald Engler, Andrea Engler, Anke Hinney, Winfried Rief, Oliver Witzke, Manfred Schedlowski

**Affiliations:** 1 Institute of Medical Psychology and Behavioral Immunobiology, University Hospital Essen, University of Duisburg-Essen, Essen, Germany; 2 Department of Nephrology, University Hospital Essen, University of Duisburg-Essen, Essen, Germany; 3 Department of Child and Adolescent Psychiatry, Psychosomatics and Psychotherapy, University Hospital Essen, Essen, Germany; 4 Division of Clinical Psychology, University of Marburg, Marburg, Germany; 5 Clinic for Anesthesiology and Intensive Care, University Hospital Essen, University of Duisburg-Essen, Essen, Germany; Sichuan University, China

## Abstract

A large number of unwanted adverse events and symptoms reported by patients in clinical trials are not caused by the drug provided, since most of adverse events also occur in corresponding placebo groups. These nocebo effects also play a major role in drug discontinuation in clinical practice, negatively affecting treatment efficacy as well as patient adherence and compliance. Experimental and clinical data document a large interindividual variability in nocebo responses, however, data on psychological, biological or genetic predictors of nocebo responses are lacking. Thus, with an established paradigm of behaviorally conditioned immunosuppressive effects we analyzed possible genetic predictors for nocebo responses. We focused on the genetic polymorphisms in the catechol-O-methyltransferase (*COMT*) gene (Val158Met) and analyzed drug specific and general side effects before and after immunosuppressive medication and subsequent placebo intake in 62 healthy male subjects. Significantly more drug-specific as well as general side effects were reported from homozygous carriers of the Val158 variant during medication as well as placebo treatment compared to the other genotype groups. Val158/Val158 carriers also had significantly higher scores in the somatosensory amplification scale (SSAS) and the BMQ (beliefs about medicine questionnaire). Together these data demonstrate potential genetic and psychological variables predicting nocebo responses after drug and placebo intake, which might be utilized to minimize nocebo effects in clinical trials and medical practice.

## Introduction

The term nocebo was created in analogy to the term placebo, and refers to the development of negative effects that are attributed to medication, albeit the drug itself does not explain the provocation of these symptoms [1], [2]. In the context of clinical trials, nocebo effects are often reported as the development of unwanted adverse events of patients allocated to the placebo groups [3]. The development of adverse side effects after placebo intake has been reported for a variety of medical conditions, and patients in the placebo arms of these clinical trials often discontinued pill intake explicitly because of symptoms that were attributed to the medication [3]–[5].

Nocebo responses, similar to placebo responses, are mediated through specific and interrelated mechanisms across different medical conditions and physiological systems [1]. Nocebo responses are steered by patient expectations towards possible unwanted side effects of a treatment or medication, which in turn can be induced by an inappropriate doctor-patient communication and/or patients information systems such as drug information leaflets [6]–[14]. In addition, associative learning and social observational learning can also play a role in the development of nocebo effects [15], [16]. These nocebo-induced side effects are not only of relevance for clinical trials, but also play a major role in drug discontinuation in clinical practice, thereby negatively affecting treatment efficacy as well as patient adherence and compliance [4]–[6], [17]–[19]. Since experimental and clinical data document a large interindividual variability in nocebo responses, one of the major challenges in this research area is to identify psychological and/or biological predictors for nocebo responses.

Genetic variation had been identified that might predict placebo responses. The Met allele of a genetic polymorphism (Val158Met) in the catechol-o-methyltransferase gene (*COMT*) was associated with increased placebo responses in patients with irritable bowel syndrome [20]. Previously, functional polymorphisms in the monoamine oxidase gene (rs6323 G-allele at *MAOA*) and the *COMT* (Met allele at Val158Met) predicted reduced placebo responses in depression [21]. However these data were not significant. Moreover, a link between polymorphisms of genes of the serotonergic system, amygdala activity and social anxiety has been reported [22]. More recently, the major degrading enzyme of endocannabinoids FAAH (fatty acid amide hydrolase) has been found to induce higher placebo analgesia for FAAH Pro129/Pro129 homozygotes [23]. However, data on genetic variables predicting nocebo responses are lacking. Psychological predictors for nocebo responses such as anticipatory anxiety for the experience of visceral pain [24], the trait pessimism for inducing unpleasant feelings after pill intake [25] and a tendency towards somatization, as well as a higher somatosensory awareness and amplification [26]–[30] have been identified.

In a model of learned immunosuppressive placebo effects in healthy humans, we were able to identify biological and psychological predictor variables for the learned inhibition in cytokine release [31]. Thus, employing this well-established paradigm of behaviorally conditioned immunosuppressive effects, the aim of the present study was to identify possible genetic predictors for nocebo responses. We focused on the *COMT* Val158Met polymorphism (rs4680) since it has been investigated most extensively [32]. The valine (Val) form catabolizes dopamine three to four-times more efficiently than the methionine (Met) form [33]. Homozygotes for the Met variant showed a more pronounced placebo response in patients with irritable bowel syndrome compared with individuals of the other genotype [20]. A possible association between reported nocebo effects and the *COMT* Val158Met polymorphism has never been investigated before.

The presented results are part of a study program on behavioral conditioning of immune functions [34]. The unique advantage of the conditioning model employed here, is the ability to analyze intra-individual nocebo responses, after intake of an immunosuppressive medication during the acquisition phase of the conditioning procedure as well as after placebo intake during the evocation phase. Psychological, immunological and neuroendocrine parameters were analyzed and drug specific and general side effects were assessed before and after medication or placebo intake respectively and the three *COMT* genotypes were analyzed with respect to their experienced side effects.

## Materials and Methods

### Ethics statement

The study was approved by the local ethics committee for human investigations of the University Hospital Essen and follows the rules stated in the Declaration of Helsinki. All participants gave written informed consent and were reimbursed for their participation.

### Subjects

As part of a larger study program on behavioral conditioning of immune functions [34] 62 healthy males of Caucasian descent (age range: 18–40 years, mean age: 25.5±0.5 years) agreed to genetic screening. All participants underwent an extensive physical and psychological assessment (self-reported questionnaires, general anamnesis and medical history). An electrocardiogram and ultrasonography of the kidneys were performed, evaluated by the physicians of the Department of Nephrology. Subjects were excluded if one of the following criteria was identified: daily intake of medication, blood donations >200 ml within the last two months, intolerance for substances (e.g. lactose) used in the study, previous participation in pharmacological studies or other medical exclusion criteria (e.g., disorders of immune or neuroendocrine system, previous or persistent mental disorders, addiction, allergies, signs of cardiovascular, hematologic or nephrologic disorders, respiratory problems or diabetes mellitus). Subjects received 500 € for their participation.

### Study design

In the established taste-immune conditioning paradigm in humans, the immunosuppressive drug cyclosporine A (CsA) (unconditioned stimulus/US) is paired with a gustatory stimulus (conditioned stimulus/CS) during acquisition. Mere re-exposition to the CS during evocation is mimicking the immunopharmacological properties of CsA, reflected by impaired Th1 cytokine production and decreased T cell proliferation [35], [36]. In order to determine the kinetics and extinction process of conditioned immunosuppression, subjects were randomly allocated into three groups.

**Figure 1:**
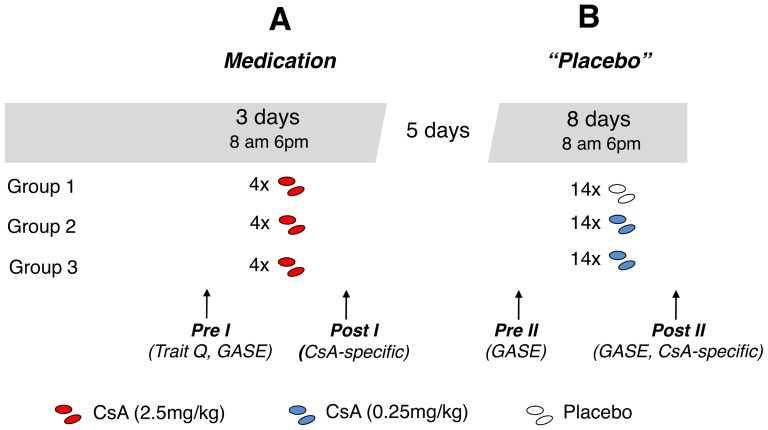
On experimental days 1 (6 pm), 2 (8 am and 6 pm) and 3 (8 am) during medication intake, all subjects in each of the 3 treatment groups received four oral doses of 2.5 mg/kg body weight of the immunosuppressive drug CsA (Sandimmun optoral, Novartis) in capsule form. In addition, subjects in groups 1 (n = 24) and 2 (n = 26) received the CS (drink) with each capsule (CsA) intake whereas subjects in group 3 (n = 12) were not exposed to the CS **(A)**. After five days wash out time, subjects received either identical looking capsules containing a placebo (lactose powder) or a subtherapeutic dose of CsA (0.25 mg/kg) fourteen times, twice a day (8 am and 6 pm respectively) with (groups 1 and 2) or without (group 3) the CS **(B)**. In order to analyze possible general treatment side effects (nocebo effects), participants were asked before the start of the study (*Pre I*), after *Medication* intake (*Post I*) as well as before (*Pre II*) and after “*Placebo*” (*Post II*) intake to fill out the GASE. Blood was drawn on the first day for baseline measurement (*Pre I*), on day 3 (*Post I*) to analyze the pharmacological effect of CsA as well as on day 8 (*Pre II*) and 15 (*Post II*) to determine possible residual effects of the drug as well as effects on physiological parameters after treatment with “*Placebo*” (sub-therapeutical doses of CsA).

On experimental days 1 (6 pm), 2 (8 am and 6 pm) and 3 (8 am) during the first week of medication intake, all subjects in all three groups received four oral doses of 2.5 mg/kg body weight of the immunosuppressive drug CsA (Sandimmun optoral, Novartis) in capsule form. In addition, subjects in groups 1 (n = 24) and 2 (n = 26) received a green-colored novel-tasting drink (150 ml strawberry milk aromatized with lavender oil) (CS) with each capsule (CsA) intake whereas subjects in group 3 (n = 12) were not exposed to the CS (drink) (Figure 1A). A pause of five days followed to allow drug wash out. During the following eight days, subjects of group 1 received identical looking capsules containing a placebo (lactose powder) together with the CS (drink). The CsA capsules were manufactured by the pharmacy of the university hospital Essen in a way so that they were not discriminable in taste and smell from the placebo capsules. To achieve this, the CsA capsules were coated with a white film of galantine and the interstitial were filled with lactose powder. Subjects in group 2 received subtherapeutic doses of CsA (0.25 mg/kg/10% of the dose employed as US) fourteen times together with the CS (twice a day; 8 am and 6 pm, respectively) whereas group 3 received the subtherapeutic dose of CsA without the drink (CS) (Figure 1B).

The subtherapeutic dose of CsA was previously determined, in which 0.25 mg/kg CsA (10% of the dose used as an US) did not significantly affect functional immunological parameters such as interleukin 2 (IL-2) secretion and mRNA expression or anti-CD3 stimulated PBMCs cytokine production [34]. Blood was drawn on the first day at 10 am for baseline measurement, on day 3 at 10 am to analyze the pharmacological effect of CsA as well as on day 8 and 15 at 10 am to determine possible residual effects of the drug. Heart rate, blood pressure, plasma catecholamines, and cortisol concentrations as well as the IL-2 concentration in culture supernatant were analyzed in parallel (Figure 1). All participants were told that the chance of receiving the immunosuppressant CsA was always 50%.

### Behavioral measures

Questionnaires assessing psychological trait variables were filled out before the first study day (Figure 1A; *Pre I*). A general medical history, traits such as beliefs about medicines (Beliefs about Medicines Questionnaire_extended version/BMQ) [37], assessing patients' beliefs about prescribed medication acquired from the personal medical history, as well as general views about medicines in six subscales (“BMQ_general harm”: Belief that medication will do harm in general, “BMQ_general overuse”: Perception how doctors use medicine and place too much emphasis in them, “BMQ_general benefit”: The belief that a beneficial treatment can be achieved through medicines, “BMQ_sensitive soma”: Sensitivity towards drug effects, “BMQ_specific necessity”: Dependence on medication, “BMQ_specific concerns”: Being concerned about drug side effects and feeling uneasy when taking drugs) and somatosensory amplification (Somatosensory Amplification Scale/SSAS) measuring a disposition to identify natural occurring somatic and visceral sensations as being very strong, unpleasant and harmful [29], [38]. In addition, the trait version of the State-Trait-Anxiety-Inventory [39], as well as physical activity [40] were analyzed. In order to exclude any participants with high depression scores, the Hospital Depression Scale [41] was also used. Additionally, subjects were asked repeatedly during *Medication* and “*Placebo*” intake to rate CsA-specific side effects on a five-point Lickert scale (heat sensation in the hands and head, nausea and discomfort in the intestine and stomach, fatigue, tingling sensation in the hands, (0/“not at all”; 4/“very intense”) (Figure 1).

Before the start of the study, subjects completed the Generic Assessment of Side Effects questionnaire (GASE) [17], assessing psychological and medical indispositions of the last seven days (Figure 1A; *Pre I*). The GASE asks for the most frequent side effects in clinical trials according to FDA statistics and it also allows to assess the attribution of symptoms to a specific drug. Subsequently, only those symptoms that were attributed to the alleged drug were measured before the first intake of placebo capsules (Figure 1A; *Pre II*), as well as after fourteen intakes of capsules containing no pharmacological agents (placebo) or a subtherapeutic dose of CsA (0.25 mg/kg) respectively, to analyze possible unwanted side effects, which were ascribed to the “*Placebo*” treatment (Figure 1B; *Post II*).

Cardiovascular parameters heart rate and systolic and diastolic blood pressure were analyzed before (*Pre I*) and after (*Post I*) *Medication* intake (acquisition phase) and before (*Pre I*) and after (*Post I*) “*Placebo*” intake (evocation phase), respectively.

### Measurement of blood concentrations of CsA

CsA concentrations in whole blood were assessed using Siemens Dimension Flex reagent cartridge (Erlangen, Germany), according to the manufacturer's instructions.

### Cell isolation

In order to determine the effects of CsA, peripheral blood mononuclear cells (PBMCs) were isolated by density gradient centrifugation (Ficoll-Paque Plus, GE Healthcare, Munich, Germany). Cells were washed with Hanks' Balanced Salt Solution (Life Technologies, Darmstadt, Germany), counted with an automated hematology analyser (KX-21 N, Sysmex Deutschland GmbH, Norderstedt, Germany) and adjusted to 5×10^6^ cells/ml in cell culture medium (RPMI 1640 supplemented with GlutaMAX I, 25 mMHepes, 10% fetal bovine serum, 50 µg/ml gentamicin; Life Technologies).

### Determination of IL-2 in culture supernatant

PBMC suspensions (5×10^6^ cells/ml) were transferred to 96-well flat bottom tissue culture plates and were stimulated with 20 ng/ml of soluble mouse anti-human CD3 monoclonal antibody (clone: HIT3a; BD Pharmingen, San Diego, CA) for 24 h (37°C, 5% CO_2_). Concentration of IL-2 in culture supernatants was quantified using a commercial ELISA (Biolegend, San Diego, USA) according to the manufacturer's instructions.

### Genotyping

Genomic DNA was extracted from whole blood using peqGOLD Blood DNA Mini Kit (PEQLAB Biotechnologie, Erlangen, Germany) according to the manufacturer's protocol. Genotyping was performed on a 7500 Fast Real-Time PCR System using the TaqMan SNP Genotyping assay for rs4680 (C_25746809_50) and the TaqMan genotyping master mix (Applied Biosystems, Darmstadt, Germany) following the manufacturer's instructions. Allelic discrimination analysis was performed with the SDS version 1.4 software (Applied Biosystems, Foster City, USA).

### Statistical analysis

All data are expressed as mean ±SEM. Immunological, neuroendocrine, cardiovascular parameters, as well as CsA levels in whole blood were analyzed using repeated-measures analysis of variance (ANOVA). Psychological characteristics as well as behavioral parameters were compared with univariate analysis of variances (ANOVA) followed by Bonferroni post-hoc tests. Pearson correlations were measured, using PASW statistics version 18 (SPSS, Chicago, USA). Allele and genotype distributions did not deviate from Hardy-Weinberg equilibrium. To analyze association of all variants, Fisher's exact test (allelic association) was calculated with PLINK [42]. If not stated otherwise, all p-values are asymptotic, two-sided and corrected for multiple testing. The significance-level was set at *p*<0.05.

## Results

In the first step we analyzed possible differences between treatment groups 1 to 3 in all variables. Subjects in these groups did not differ in any of the sociodemographic, psychological, cardiovascular (data not shown) or immunological parameters analyzed here (Tables 1 and 2). In addition, subjects of all three groups did not significantly differ in their perceived psychological and medical indispositions of the last seven days documented with the GASE before study entry (baseline; F = 0.05, n.s.), as well as in their reported general (F = 1.34, n.s.) and CsA-specific (F = 1.34, n.s.) side effects during *Medication* intake (Figure 1, *Post I*). Moreover, during “*Placebo*” treatment, no significant group differences were observed, neither in reported general side effects (GASE; *Post II* (F = 0.51, n.s.); nor CsA-specific side effects (F = 0.36, n.s.) (Table 3).

**Table 1:** Sociodemographic and psychological characteristics of the three experimental groups.

Treatment group	Group 1 (n = 24)	Group 2 (n = 26)	Group 3 (n = 12)
Age (years)	25.0±0.7	25.9±0.9	25.5±0.9
Body mass index (kg/m^2^)	22.5±1.1	22.3±1.4	24.2±1.0
Physical activity (FfkA)	42.3±6.8	37.2±5.3	51.5±9.3
Trait anxiety (STAI)	33.1±1.3	37.3±1.8	36.3±1.8
SSAS	24.8±1.1	25.9±1.1	27.1±1.4
BMQ_general harm	9.8±0.6	9.2±0.7	10.5±0.7
BMQ_general overuse	13.7±0.5	12.9±0.6	13,7±0.7
BMQ_general benefit	15.7±0.5	16.4±0.5	15.6±0.7
BMQ_sensitive soma	7.7±0.6	8.3±0.7	9.6±1.0
BMQ_specific necessity	7.6±0.4	7.8±0.5	8.1±0.8
BMQ_specific concerns	9.6±0.7	9.2±0.9	11.3±1.1

Age, body mass index, physical activity, trait anxiety (STAI), SSAS, BMQ_ general harm, BMQ_general overuse, BMQ_general benefit, BMQ_sensitive soma, BMQ_specific necessity and BMQ_specific were compared between all three treatment groups using univariate ANOVA. Groups did not significantly differ in any of the variables listed (all p>0.05). Data are shown as mean ±SEM.

**Table 2:** CsA serum levels and IL-2 protein concentrations of the three treatment groups during *Medication* and “*Placebo*” intake.

	Group	*Medication*		“*Placebo*”	
		*Pre I*	*Post I*	*Pre II*	*Post II*
**CsA levels in whole blood** (*ng/ml*)	Group 1	n.d.	1285.8±41.3 *	n.d.	n.d.
	Group 2	n.d.	1096.1±94.0 *	n.d.	60.4±5.5 *
	Group 3	n.d.	1482.3±77.6 *	n.d.	81.9±6.5 *
**IL-2 in culture supernatant** (*pg/ml*)	Group 1	488.4±76.4	128.3±18.3 *	452.2±68.8	432.5±75.8
	Group 2	350.5±78.2	115.7±22.2 *	341.6±60.9	303.0±54.5
	Group 3	278.4±43.9	164.4±31.6 *	378.6±60.8	492.8±83.5

CsA treatment during *Medication* significantly increased CsA serum levels and significantly suppressed IL-2 protein concentrations after anti-CD3 stimulation in all groups. During the “*Placebo*” condition, treatment with subtherapeutical doses of CsA slightly increased CsA levels (groups 2 and 3), however did not effect IL-2 concentration in these groups. (ANOVA, time effect; *p<0.001) (n.d. =  not detectable). Data are shown as mean ±SEM.

**Table 3:** CsA-specific and general side effects during the *Medication* and “*Placebo*” condition.

		*Medication*		“*Placebo*”	
Group	General side effects (GASE) Baseline	CsA-specific side effects	General side effects (GASE)	CsA-specific side effects	General side effects (GASE)
Group 1	5.0±1.2	7.1±1.3	6.3±1.1	13.0±3.5	1.0±0.4
Group 2	4.7±1.4	8.0 ±1.6	5.9±1.7	18.7±5.4	1.2±0.4
Group 3	4.3±1.2	12.1±3.9	9.9±3.7	19.5±11.5	2.3±2.1

Treatment groups did not significantly differ in reported CsA-specific and general side effects analyzed with the GASE, neither before study participation (GASE Baseline), nor during the *Medication* and the “*Placebo*” condition (all p>0.05). Data are shown as mean ±SEM.

### Genotyping and analyses of general and CsA-specific side effects

**Figure 2:**
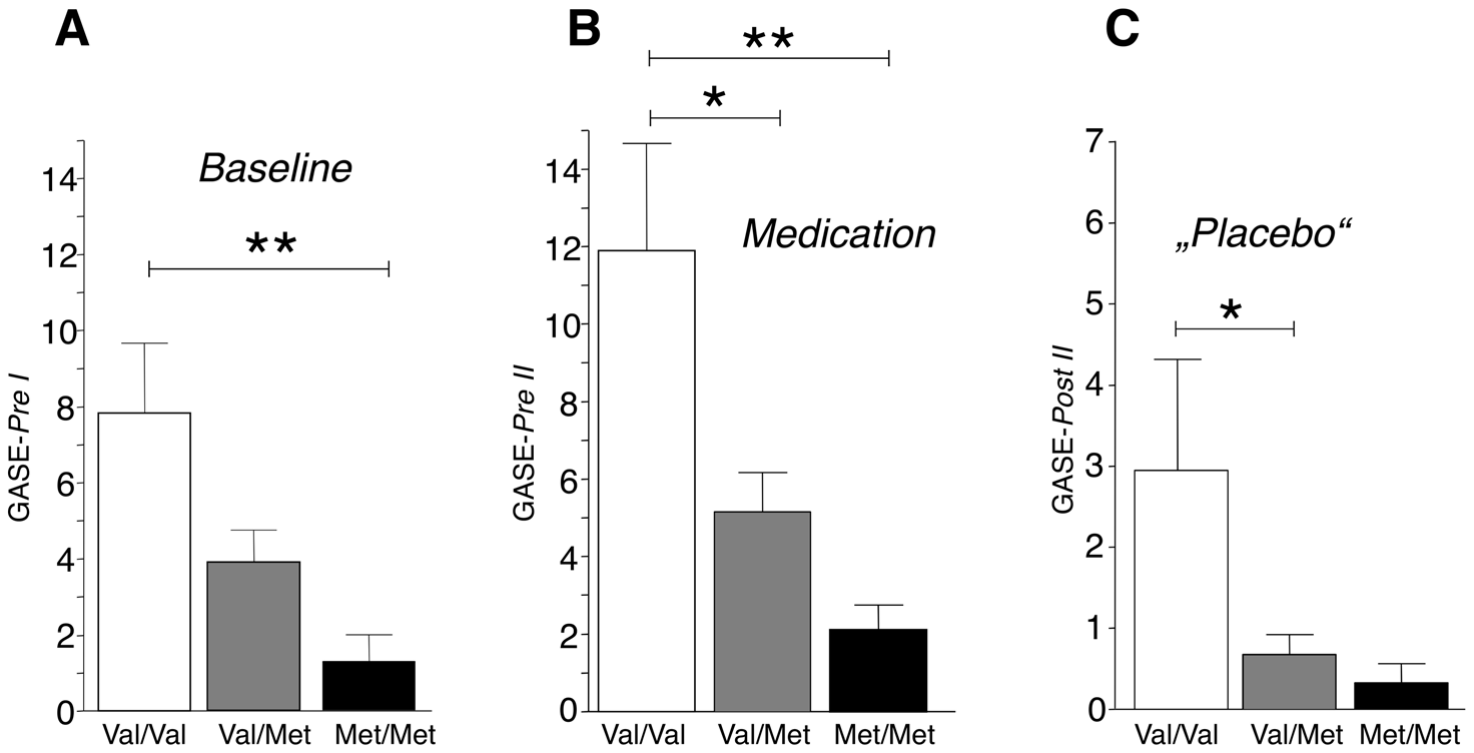
General psychological and medical indispositions were analyzed with the GASE before study entry (*Baseline*) (A), after *Medication* intake (B) and “*Placebo*” intake (C), respectively. Homozygous Val158 carriers experienced significantly more general psychological and medical indispositions before study entry and after four medication intakes **(B)**. The Val/Val group also showed the strongest nocebo response, reflected by most reported side effects after the “*Placebo*” intake **(C)**. Bars represent mean ±SEM; data were analyzed with univariate ANOVAs. In case of significant F tests, these were followed by Bonferroni post hoc tests; *p<0.05, **p<0.001.

After confirming that treatment groups showed no significant differences in reported side effects, volunteers were then compared according to the respective three genotype groups: Homozygotes for the Val158 allele, heterozygotes (Val/Met), and homozygotes for the Met158 allele. Genotyping revealed genotype frequencies of 30.7% (Val/Val; n = 19), 54.8% (Val/Met; n = 34) and 14.5% (Met/Met, n = 9) for the *COMT* Val158Met polymorphism. Subjects of the three treatment groups were evenly distributed across the three genotype groups. Subsequent analyses revealed significant differences between allele carriers of the *COMT* Val158Met polymorphism and their experienced side effects. When analyzing general psychological and medical indispositions with the GASE questionnaire before study entry (*Baseline*, *Pre I*), Val158/Val158 homozygous carriers reported significantly more general psychological and medical complaints compared to the Met158/Met158 and Val158/Met158 groups (F = 4.6; p<0.01) (Fig. 2A). In parallel with these findings this group also reported significantly more general psychological and medical indispositions (GASE) after *Medication* intake (F = 5.9; p<0.01) compared to both other genotypes (Fig. 2B). The identical pattern occurred when subjects were asked after “*Placebo*” intake. Again, the Val158/Val158 homozygous carriers reported more experienced general side effects compared to all other genotypes (F = 4.7; p<0.02) (Fig. 2C).

**Figure 3:**
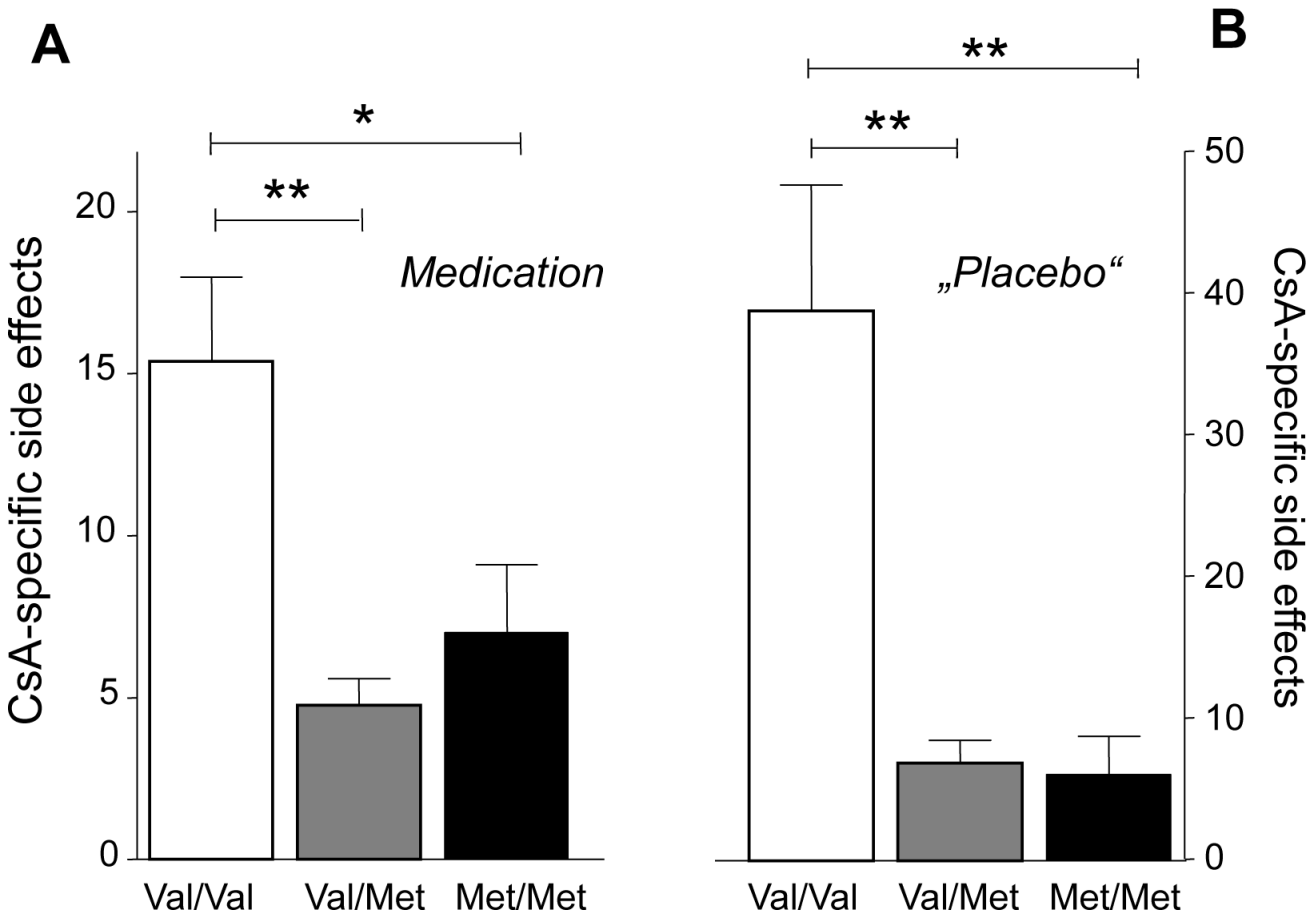
Reported CsA-specific side effects after *Medication* (A) and “*Placebo*” intake, respectively (B). Significantly higher CsA-specific side effects, after four medication intakes, were reported by homozygous Val158/Val158 carriers (**A**). This difference was even more pronounced after fourteen “*Placebo*” intakes (**B**). Bars represent mean ±SEM. In case of significant F tests, these were followed by Bonferroni post hoc tests; *p<0.05, **p<0.001.

The differences in reported general side effects between allele carriers of the COMT Val158Met polymorphism were paralleled by significant group differences in the experienced CsA-specific side effects. After *Medication* intake, ANOVAs showed significant differences for CsA-specific side effects (F = 11.9; p<0.001) with significant more side effects reported in Val158/Val158 compared to the Val158/Met158 (p<0.001) and Met158/Met158 groups (p<0.05) (Figure 3A). Similar differences in reported CsA-specific side effects between allel carriers were observed during “*Placebo*” treatment (F = 13.1; p<0.001) with the most pronounced side effects reported in the Val/Val homozygous carriers compared to the Val158/Met158 (p<0.001) and the Met158 homozygous (p<0.01) groups (Figure 3B).

### Genotyping and trait psychological parameters

Univariate ANOVAs showed no significant differences between the three genotypes in sociodemographic variables or trait anxiety (Table 4). In contrast however, homozygous carriers of the Val158 allele showed a significantly higher score in the Somatosensory Amplification Scale (SSAS) (F = 8.8, p<0.001) compared to the other genotypes (heterozygotes p<0.001; Met158/Met158 homozygous carriers (p<0.01), reflecting a disposition to identify natural occurring somatic and visceral sensations as being very strong, unpleasant and harmful. In addition, homozygous carriers of the Val158 scored significantly higher compared to both other genotypes within four subcales of the BMQ, indicating a significantly higher belief that medication will harm in general and a more sensitive reaction towards their effects (Table 4). They were significantly more convinced that medication harms them in general (BMQ_general harm; F = 4.2; p<0.05) and react more sensitive towards their effects (BMQ_sensitive soma; F = 13.14; p<0.001). Val158/Val158 homozygotes also view themselves as significantly more dependent on medication (BMQ_specific necessity; F = 5.6; p<0.01) and at the same time are more concerned about their side effects and feel uneasy when taking medication (BMQ_specific concerns; F = 6.3; p<0.01). The three genotype groups did not significantly differ in the two BMQ subscales “general overuse” and “general benefit” (Table 4). In order to exclude subjects with high scores in the Hospital Depression Scale (HADS) mean scores and standard errors were calculated for all participants, which revealed no unusually high scores (3.5±0.34).

**Table 4:** Differences in the somatosensory amplification scale (SSAS) and four subscales of the Beliefs about Medicines Questionnaire (BMQ) depending on the respective genotype group.

Genotype	Val158/Val158 (n = 19)	Val158/Met158 (n = 34)	Met158/Met158 (n = 9)
Age (years)	25.7±1.1	25.3±0.6	25.6±1.3
Body mass index (kg/m^2^)	21.5±1.9	23.7±0.5	22.0±2.9
Physical activity (FfkA)	46.6±8.7	38.2±4.2	46.0±11.2
Trait anxiety (STAI)	38.5±1.8	34.4±1.3	33.3±2.6
SSAS	29.4±1.3 **	24.3±0.8	22.9±1.3
BMQ_general harm	11.0±0.7 *	8.8±0.5	10.6±0.7
BMQ_general overuse	14.1±0.7	12.9±0.5	13.7±0.8
BMQ_general benefit	15.7±0.6	16.0±0.4	16.0±0.7
BMQ_sensitive soma	11.0±0.8 ***	7.4±0.4	6.2±0.5
BMQ_specific necessity	9.2±0.6 *	7.2±0.4	6.8±0.5
BMQ_specific concerns	12.1±1.1 **	9.1±0.6	7.4±0.7

Age, body mass index, physical activity, trait anxiety (STAI), SSAS, BMQ_general harm, BMQ_general overuse, BMQ_general benefit, BMQ_sensitive soma, BMQ_specific necessity and BMQ_specific concerns were compared among COMT genotype groups (Val158/Val158, n = 19; Val158/Met158, n = 34; Met158/Met158, n = 9). Data were analyzed using univariate ANOVA. In case of significant F tests, these were followed by Bonferroni post hoc tests. Data are shown as mean ± SEM; *p<0.05, **p<0.01, ***p<0.001.

### CsA levels, immunological and cardiovascular parameters

CsA levels were determined two hours after the last of four CsA intakes (10 am; peak level) and after the *medication* intake condition. CsA concentrations were significantly increased in all three genotype subgroups without significant differences between groups (Table S1). After treatment with subtherapeutic CsA doses, marginal CsA concentrations could be detected in peripheral blood with no differences between groups. In addition, ANOVA did not show significant differences between the three genotypes at the *COMT* Val158Met polymorphism in cardiovascular parameters (data not shown), CsA or IL-2 levels analyzed during the treatment conditions (Table S1). Thus, the significantly increased reported specific and general treatment side effects in Val158/Val158 homozygous carriers during drug (*Medication* intake) and “*Placebo*” treatment is not due to distinct physiological responses to the treatments.

## Discussion

Clinical and experimental data on psychological, biological or genetic variables predicting placebo responses are rare; data on genetic variables predicting nocebo responses are lacking. With an established model of learned immunosuppression we focused on the catechol-O-methyltransferase (*COMT*) Val158Met polymorphism and analyzed an association of reported drug-specific and general side effects during medication and placebo intake of the *COMT* genotypes. Our results demonstrate significantly more drug-specific as well as general side effects in homozygous Val158 carriers during medication and placebo treatment compared to the other genotypes. These differences in nocebo responses were not due to treatment specific changes in psychological (anxiety) or biological (cardiovascular, immunological) parameters since allele carriers did not differ in these variables. In addition, homozygous Val158/Val158 carriers displayed significantly higher scores in the somatosensory amplification scale (SSAS) and the BMQ (beliefs about medicine questionnaire) compared to the other genotype groups.

Together these data suggest that *COMT* Val158Met, specifically the Val158/Val158 genotype, is a potential genetic marker for nocebo responders. This is primarily true for the specific model employed here, in which healthy male subjects received a short-term treatment during the *Medication* period with an immunosuppressive drug, the calcineurin inhibitor CsA. Whether and to what extent this is a generalizable phenomenon and transferable to other drugs and/or patient populations needs to be investigated [20]–[22], [43]. However, the Val158/Val158 group reported the most pronounced CsA-specific as well as general side effects analyzed with the GASE scale not only during medication intake. These genotypes also reported twice as many specific side effects after taking fourteen placebo capsules during the “*Placebo*” treatment compared to the intake of the drug CsA. In addition, Val158/Val158 homozygous carriers showed a significantly more pronounced disposition to identify natural occurring somatic and visceral sensations as being very strong, unpleasant and harmful, which was measured with the SSAS. These results seemed to be independent of treatment-specific effects during medication or placebo intake, since homozygous and heterozygous Val158 allele carriers did not differ with respect to their drug levels, cardiovascular or immunological parameters analyzed before and after *Medication* or “*Placebo*” condition. Thus, together with the data showing that the *COMT* polymorphism predicted placebo responses in patients with irritable bowel syndrome and depression [44], [45], these observations militate for a role of *COMT* in nocebo responses also for other physiological systems and diseases. Please note that the Met158 allele was associated with increased placebo responses in patients with irritable bowel syndrome [20]. So potentially both *COMT* alleles display opposite sides of the coin pertaining to placebo/nocebo response.

The reason for the increased sensitivity for treatment specific and general side effects in the Val158/Val158 homozygotes observed in this study is unclear. The non-synonymous single nucleotide polymorphism at *COMT* (Val158Met; rs4680 [46]) leads to a functional consequence. COMT harboring Val158 catabolizes dopamine three to four-times more effectively than the Met158 form [33]. This leads to significantly lower concentrations of prefrontal dopamine in Val158/Val158 carriers compared to the other genotypes. This different availability of prefrontal dopamine seems to affect reward and information seeking behavior [47], [48] in general, but also predicted placebo responses in patients with irritable bowel syndrome, with more pronounced placebo responses in patients carrying the Met158 allele homozygously [20], [44]. A possible relationship between a smaller amount of available dopamine in the prefrontal cortex (Val158/Val158 individuals) and a more pronounced nocebo response was also reported in a study with cancer patients, where patients of the Val158/Val158 genotype experienced more pain than the other two genotypes and required higher doses of morphine [43]. Under experimental conditions however, Met158/Met158 individuals reported a higher sensitivity towards experimental pain [49], [50].

In order to ensure that higher rates of nocebo effects measured in Val158/Val158 homozygotes are not caused through another factor, we carefully controlled a range of possible interfering variables. These measures revealed that participants did not differ in sociodemographic variables, as well as psychological characteristics, which could have affected an increased experience of side effects, such as trait anxiety and physical characteristics (physical activity, BMI). Additionally, there were no group differences in cardiovascular parameters which could have been indicators of an increased stress reaction to the experimental procedure or medication intake. Moreover, CsA levels in whole blood as well as IL-2 concentrations in culture supernatant, as an indicator of the immunosuppressive effect of CsA, were monitored with no difference between the three genotype groups which could have explained the increased CsA-specific and general side effects.

The ability to analyze intra-individual nocebo responses after intake of an immunosuppressive medication during the acquisition phase of the conditioning procedure as well as after placebo intake during the evocation phase is certainly a unique advantage of the model employed here. However, there are also a number of limitations within this study. Firstly, our findings are limited due to the small number of volunteers included in the genetic analyses, which only included young and healthy males and have to be thus interpreted with caution. The sample size is not sufficient to detect small sized effects, which may bear the risk of type 2 errors, i.e., the risk to miss existing group differences due to low statistical power. Future studies in larger samples should consider multiple factor statistical models of potentially moderating or even mediating effects of sociodemographic or additional psychological or genetic variables. Secondly, the information material and systematic procedure of analyzing adverse side effects could have let to an increased number of reported side effects, compared to a “free recall” procedure, as the documentation itself is affecting the occurrence of side effects [6]. Subjects in this experiment were regularly informed about common CsA side effects and also received information material, as well as a standard questionnaire for specific and general side effects (GASE). Lastly, further psychological characteristics such as neuroticism, coping style, personality, as well as decision making should be included in future studies, in order to detect their potential effects on the response towards medication.

In summary, the identification of psychobiological and/or genetic predictor variables in order to minimize nocebo effects is of essential relevance for clinical practice and trials. Future studies have to confirm the reported personality and genetic predictor variables for nocebo responses for different drugs, different physiological systems and end organ functioning. If nocebo responders could be conveniently classified by genetic or psychological predictor variables, these individuals could receive a “personalized treatment”, such as the usage of a “contextualized informed consent” [51]. The recognition of placebo and nocebo responders will be invaluable for estimating the real drug effects, as placebo responders will contribute to an underestimation of drug effects, whereas nocebo responders will lead to an overestimation of adverse unwanted side effects.

## Supporting Information

Table S1 **CsA serum levels and IL-2 protein concentrations during the** ***Medication*** **and “** ***Placebo*** **” condition of the COMT genotype groups.** CsA treatment during *Medication* significantly increased CsA serum levels and significantly suppressed IL-2 protein concentrations after anti-CD3 stimulation in all COMT genotype groups. During the “*Placebo*” condition, treatment with subtherapeutical doses of CsA slightly increased CsA levels in Val158/Val158, Val158/Met158 as well as Met158/Met158 allel carriers, however did not significantly affect IL-2 concentrations in these groups. (ANOVA, *p<0.001, time effect) (n.d. =  not detectable). Data are shown as mean ± SEM.
(DOCX)

## References

[1] Pacheco-López G, Engler H, Niemi M-B, Schedlowski M (2006) Expectations and associations that heal: Immunomodulatory placebo effects and its neurobiology. Brain Behav Immun 20: 430–446. 16887325 10.1016/j.bbi.2006.05.003

[2] Rief W, Hofmann SG, Nestoriuc Y (2008) The Power of Expectation – Understanding the Placebo and Nocebo Phenomenon. Soc Personal Psychol Compass 2: 1624–1637.

[3] Mitsikostas DD, Mantonakis LI, Chalarakis NG (2011) Nocebo is the enemy, not placebo. A meta-analysis of reported side effects after placebo treatment in headaches. Cephalalgia 31: 550–561. 21216874 10.1177/0333102410391485

[4] Enck P, Bingel U, Schedlowski M, Rief W (2013) The placebo response in medicine: minimize, maximize or personalize? Nat Rev Drug Discov 12: 191–204. 23449306 10.1038/nrd3923

[5] Rief W, Bingel U, Schedlowski M, Enck P (2011) Mechanisms involved in placebo and nocebo responses and implications for drug trials. Clin Pharmacol Ther 90: 722–726. 21975346 10.1038/clpt.2011.204

[6] Rief W, Avorn J, Barsky AJ (2006) Medication-attributed adverse effects in placebo groups: implications for assessment of adverse effects. Arch Intern Med 166: 155–160. 16432082 10.1001/archinte.166.2.155

[7] Mondaini N, Gontero P, Giubilei G, Lombardi G, Cai T, et al (2007) Finasteride 5 mg and sexual side effects: how many of these are related to a nocebo phenomenon? J Sex Med 4: 1708–1712. 17655657 10.1111/j.1743-6109.2007.00563.x

[8] Daniels AM, Sallie R (1981) Headache, lumbar puncture, and expectation. Lancet 1: 1003. 10.1016/s0140-6736(81)91771-2 6112373

[9] Silvestri A, Galetta P, Cerquetani E, Marazzi G, Patrizi R, et al (2003) Report of erectile dysfunction after therapy with beta-blockers is related to patient knowledge of side effects and is reversed by placebo. Eur Heart J 24: 1928–1932. 14585251 10.1016/j.ehj.2003.08.016

[10] Myers MG, Cairns JA, Singer J (1987) The consent form as a possible cause of side effects. Clin Pharmacol Ther 42: 250–253. 3621780 10.1038/clpt.1987.142

[11] Varelmann D, Pancaro C, Cappiello EC, Camann WR (2010) Nocebo-induced hyperalgesia during local anesthetic injection. Anesth Analg 110: 868–870. 20042440 10.1213/ANE.0b013e3181cc5727

[12] Lang EV, Hatsiopoulou O, Koch T, Berbaum K, Lutgendorf S, et al (2005) Can words hurt? Patient-provider interactions during invasive procedures. Pain 114: 303–309. 15733657 10.1016/j.pain.2004.12.028

[13] O'Connor AM, Pennie RA, Dales RE (1996) Framing effects on expectations, decisions, and side effects experienced: the case of influenza immunization. J Clin Epidemiol 49: 1271–1276. 8892495 10.1016/s0895-4356(96)00177-1

[14] Colloca L, Finniss D (2012) Nocebo effects, patient-clinician communication, and therapeutic outcomes. JAMA 307: 567–568. 22318275 10.1001/jama.2012.115 PMC6909539

[15] Swider K, Bąbel P (2013) The effect of the sex of a model on nocebo hyperalgesia induced by social observational learning. Pain 154: 1312–1317. 23725779 10.1016/j.pain.2013.04.001

[16] Vögtle E, Barke A, Kröner-Herwig B (2013) Nocebo hyperalgesia induced by social observational learning. Pain 154: 1427–1433. 23707275 10.1016/j.pain.2013.04.041

[17] Rief W, Barsky AJ, Glombiewski JA, Nestoriuc Y, Glaesmer H, et al (2011) Assessing general side effects in clinical trials: reference data from the general population. Pharmacoepidemiol Drug Saf 20: 405–415. 21442687 10.1002/pds.2067

[18] Häuser W, Hansen E, Enck P (2012) Nocebo phenomena in medicine: their relevance in everyday clinical practice. Dtsch Arztebl Int 109: 459–465. 22833756 10.3238/arztebl.2012.0459 PMC3401955

[19] Benedetti F, Amanzio M (2011) The placebo response: how words and rituals change the patient's brain. Patient Educ Couns 84: 413–419. 21621366 10.1016/j.pec.2011.04.034

[20] Hall KT, Lembo AJ, Kirsch I, Ziogas DC, Douaiher J, et al (2012) Catechol-O-methyltransferase val158met polymorphism predicts placebo effect in irritable bowel syndrome. PLoS ONE 7: e48135. 23110189 10.1371/journal.pone.0048135 PMC3479140

[21] Leuchter AF, McCracken JT, Hunter AM, Cook IA, Alpert JE (2009) Monoamine oxidase a and catechol-o-methyltransferase functional polymorphisms and the placebo response in major depressive disorder. J Clin Psychopharmacol 29: 372–377. 19593178 10.1097/JCP.0b013e3181ac4aaf

[22] Furmark T, Appel L, Henningsson S, Ahs F, Faria V, et al (2008) A link between serotonin-related gene polymorphisms, amygdala activity, and placebo-induced relief from social anxiety. J Neurosci 28: 13066–13074. 19052197 10.1523/JNEUROSCI.2534-08.2008 PMC6671592

[23] Peciña M, Martínez-Jauand M, Hodgkinson C, Stohler CS, Goldman D, et al (2014) FAAH selectively influences placebo effects. Mol Psychiatry 19: 385–391. 24042479 10.1038/mp.2013.124 PMC4222079

[24] Elsenbruch S, Schmid J, Bäsler M, Cesko E, Schedlowski M, et al (2012) How positive and negative expectations shape the experience of visceral pain: an experimental pilot study in healthy women. Neurogastroenterol Motil 24: 914–e460. 22650270 10.1111/j.1365-2982.2012.01950.x

[25] Geers AL, Helfer SG, Kosbab K, Weiland PE, Landry SJ (2005) Reconsidering the role of personality in placebo effects: dispositional optimism, situational expectations, and the placebo response. J Psychosom Res 58: 121–127. 15820839 10.1016/j.jpsychores.2004.08.011

[26] Wilson L, Dworkin SF, Whitney C, LeResche L (1994) Somatization and pain dispersion in chronic temporomandibular disorder pain. Pain 57: 55–61. 8065797 10.1016/0304-3959(94)90107-4

[27] Davis C, Ralevski E, Kennedy SH, Neitzert C (1995) The role of personality factors in the reporting of side effect complaints to moclobemide and placebo: a study of healthy male and female volunteers. J Clin Psychopharmacol 15: 347–352. 8830066 10.1097/00004714-199510000-00007

[28] Joyce CR (1959) Consistent differences in individual reactions to drugs and dummies. Br J Pharmacol Chemother 14: 512–521. 14408028 10.1111/j.1476-5381.1959.tb00958.x PMC1481910

[29] Barsky AJ, Wyshak G, Klerman GL (1990) The somatosensory amplification scale and its relationship to hypochondriasis. J Psychiatr Res 24: 323–334. 2090830 10.1016/0022-3956(90)90004-a

[30] Barsky AJ, Orav EJ, Ahern DK, Rogers MP, Gruen SD, et al (1999) Somatic style and symptom reporting in rheumatoid arthritis. Psychosomatics 40: 396–403. 10479944 10.1016/s0033-3182(99)71204-1

[31] Ober K, Benson S, Vogelsang M, Bylica A, Günther D, et al (2012) Plasma noradrenaline and state anxiety levels predict placebo response in learned immunosuppression. Clin Pharmacol Ther 91: 220–226. 22166852 10.1038/clpt.2011.214

[32] Grossman MH, Emanuel BS, Budarf ML (1992) Chromosomal mapping of the human catechol-O-methyltransferase gene to 22q11.1----q11.2. Genomics 12: 822–825. 1572656 10.1016/0888-7543(92)90316-k

[33] Lachman HM, Papolos DF, Saito T, Yu YM, Szumlanski CL, et al (1996) Human catechol-O-methyltransferase pharmacogenetics: description of a functional polymorphism and its potential application to neuropsychiatric disorders. Pharmacogenetics 6: 243–250. 8807664 10.1097/00008571-199606000-00007

[34] Albring A, Wendt L, Benson S, Nissen S, Yavuz Z, et al. (2014) Preserving learned immunosuppressive placebo response: Perspectives for clinical application. Clin Pharmacol Ther (*In press*). 10.1038/clpt.2014.75 24699032

[35] Wirth T, Ober K, Prager G, Vogelsang M, Benson S, et al (2011) Repeated recall of learned immunosuppression: evidence from rats and men. Brain Behav Immun 25: 1444–1451. 21645613 10.1016/j.bbi.2011.05.011

[36] Goebel MU, Trebst AE, Steiner J, Xie YF, Exton MS, et al (2002) Behavioral conditioning of immunosuppression is possible in humans. FASEB J 16: 1869–1873. 12468450 10.1096/fj.02-0389com

[37] Horne R, Weinman J, Hankins M (1999) The beliefs about medicines questionnaire: The development and evaluation of a new method for assessing the cognitive representation of medication. Psychol Health 14: 1–24.

[38] Barsky AJ, Goodson JD, Lane RS, Cleary PD (1988) The amplification of somatic symptoms. Psychosom Med 50: 510–519. 3186894 10.1097/00006842-198809000-00007

[39] Kendall PC, Finch AJ Jr, Auerbach SM, Hooke JF, Mikulka PJ (1976) The State-Trait Anxiety Inventory: a systematic evaluation. J Consult Clin Psychol 44: 406–412. 932270 10.1037//0022-006x.44.3.406

[40] Frey I, Berg A, Grathwohl D, Keul J (1999) Freiburger Fragebogen zur körperlichen Aktivität- Entwicklung, Prüfung und Anwendung. Soz Praventiv Med 44: 55–64. 10.1007/BF01667127 10407953

[41] Zigmond AS, Snaith RP (1983) The hospital anxiety and depression scale. Acta Psychiatr Scand 67: 361–370. 6880820 10.1111/j.1600-0447.1983.tb09716.x

[42] Purcell S, Neale B, Todd-Brown K, Thomas L, Ferreira MAR, et al (2007) PLINK: a tool set for whole-genome association and population-based linkage analyses. Am J Hum Genet 81: 559–575. 17701901 10.1086/519795 PMC1950838

[43] Rakvåg TT, Klepstad P, Baar C, Kvam T-M, Dale O, et al (2005) The Val158Met polymorphism of the human catechol-O-methyltransferase (COMT) gene may influence morphine requirements in cancer pain patients. Pain 116: 73–78. 15927391 10.1016/j.pain.2005.03.032

[44] Hall KT, Kaptchuk TJ (2013) Genetic biomarkers of placebo response: what could it mean for future trial design? Clin Investig (Lond) 3: 311–314. 10.4155/cli.13.8 PMC3774308 24049631

[45] Wray NR, James MR, Dumenil T, Handoko HY, Lind PA, et al (2008) Association study of candidate variants of COMT with neuroticism, anxiety and depression. Am J Med Genet B Neuropsychiatr Genet 147B: 1314–1318. 18384078 10.1002/ajmg.b.30744

[46] Lotta T, Vidgren J, Tilgmann C, Ulmanen I, Melén K, et al (1995) Kinetics of human soluble and membrane-bound catechol O-methyltransferase: a revised mechanism and description of the thermolabile variant of the enzyme. Biochemistry 34: 4202–4210. 7703232 10.1021/bi00013a008

[47] Lancaster TM, Linden DE, Heerey EA (2012) COMT val158met predicts reward responsiveness in humans. Genes Brain Behav 11: 986–992. 22900954 10.1111/j.1601-183X.2012.00838.x

[48] Doll BB, Hutchison KE, Frank MJ (2011) Dopaminergic genes predict individual differences in susceptibility to confirmation bias. J Neurosci 31: 6188–6198. 21508242 10.1523/JNEUROSCI.6486-10.2011 PMC3098533

[49] Zubieta J-K, Heitzeg MM, Smith YR, Bueller JA, Xu K, et al (2003) COMT val158met genotype affects mu-opioid neurotransmitter responses to a pain stressor. Science 299: 1240–1243. 12595695 10.1126/science.1078546

[50] Jensen KB, Lonsdorf TB, Schalling M, Kosek E, Ingvar M (2009) Increased sensitivity to thermal pain following a single opiate dose is influenced by the COMT val(158)met polymorphism. PLoS ONE 4: e6016. 19547755 10.1371/journal.pone.0006016 PMC2695541

[51] Wells RE, Kaptchuk TJ (2012) To tell the truth, the whole truth, may do patients harm: the problem of the nocebo effect for informed consent. Am J Bioeth 12: 22–29. 10.1080/15265161.2011.652798 PMC3352765 22416745

