# Supplementary material for: Catechol-O-Methyltransferase Val158Met Polymorphism Is Associated with Somatosensory Amplification and Nocebo Responses
Source: PLoS One. 2014 Sep 15;9(9):e107665. doi: 10.1371/journal.pone.0107665 (PMC4164653; doi:10.1371/journal.pone.0107665)
Supplement: Table S1 — CsA serum levels and IL-2 protein concentrations during the Medication and “ Placebo ” condition of the COMT genotype groups. CsA treatment during Medication significantly increased CsA serum levels and significantly suppressed IL-2 protein concentrations after anti-CD3 stimulation in all COMT genotype groups. During the “Placebo” condition, treatment with subtherapeutical doses of CsA slightly increased CsA levels in Val158/Val158, Val158/Met158 as well as Met158/Met158 allel carriers, however did not significantly affect IL-2 concentrations in these groups. (ANOVA, *p<0.001, time effect) (n.d. = not detectable). Data are shown as mean ± SEM. (DOCX) [file pone.0107665.s001.docx]

**Table S1**

CsA serum levels and IL-2 protein concentrations during the *Medication* and “*Placebo*” condition of the COMT genotype groups.

|  | **Group** | ***Medication*** | |  | **“*Placebo*”** | |
| --- | --- | --- | --- | --- | --- | --- |
|  |  | ***Pre I*** | **Post I** |  | ***Pre II*** | **Post II** |
| **CsA levels in whole blood**  *(ng/ml)* | Val/Val | n.d. | 1315.4 ± 61.4 * |  | n.d. | 62.1 ± 7.6 * |
|  | Val/Met | n.d. | 1209.9 ± 65.9 * |  | n.d. | 48.5 ± 5.3 * |
|  | Met/Met | n.d. | 1340.2 ± 110.3 * |  | n.d. | 49.9 ± 9.4 * |
|  |  |  |  |  |  |  |
| **IL-2 in culture supernatant**  *(pg/ml)* | Val/Val | 375.4 ± 61.0 | 160.4 ± 23.9 * |  | 395.4 ± 61.0 | 393.5 ± 60.3 |
|  | Val/Met | 393.4 ± 63.6 | 127.4 ± 19.9 * |  | 375.9 ± 54.0 | 402.8 ± 59.6 |
|  | Met/Met | 169.3 ± 42.4 | 75.4 ± 18.7 * |  | 398.6 ± 115.3 | 300.0 ± 103.8 |
|  |  |  |  |  |  |  |

CsA treatment during *Medication* significantly increased CsA serum levels and significantly suppressed IL-2 protein concentrations after anti-CD3 stimulation in all COMT genotype groups. During the “*Placebo*” condition, treatment with subtherapeutical doses of CsA slightly increased CsA levels in Val158/Val158, Val158/Met158 as well as Met158/Met158 allel carriers, however did not significantly affect IL-2 concentrations in these groups. (ANOVA, **p*<0.001, time effect) (n.d.= not detectable). Data are shown as mean ± SEM.
